# Rapid detection of an I38T amino acid substitution in influenza polymerase acidic subunit associated with reduced susceptibility to baloxavir marboxil

**DOI:** 10.1111/irv.12728

**Published:** 2020-02-16

**Authors:** Mina Nakauchi, Emi Takashita, Seiichiro Fujisaki, Masayuki Shirakura, Rie Ogawa, Hiroko Morita, Hideka Miura, Shinji Saito, Shinji Watanabe, Takato Odagiri, Tsutomu Kageyama

**Affiliations:** ^1^ Influenza Virus Research Center National Institute of Infectious Diseases Tokyo Japan

**Keywords:** antiviral, baloxavir marboxil, diagnosis, influenza, polymerase acidic subunit, RNase H2‐dependent PCR

## Abstract

**Background:**

The novel cap‐dependent endonuclease inhibitor baloxavir marboxil was approved in February 2018 for the treatment of influenza virus infection in Japan. In vitro studies have revealed that an I38T substitution in the polymerase acidic subunit (PA) is associated with reduced susceptibility of influenza viruses to baloxavir.

**Objectives:**

Development of a rapid and simple method for monitoring influenza A(H1N1)pdm09, A(H3N2), and B viruses possessing the I38T substitution in PA.

**Methods:**

Three assays were developed based on RNase H2‐dependent PCR (rhPCR) and named A/H1pdm PA_I38T rhPCR, A/H3 PA_I38T rhPCR, and B PA_I38T rhPCR. The assays were evaluated using cDNAs synthesized from in vitro‐transcribed *PA* gene RNA controls, RNAs purified from viruses isolated in the 2017/2018 and 2018/2019 influenza seasons, and RNAs purified from clinical specimens collected in the 2018/2019 influenza season.

**Results:**

The assays developed in this study accurately discriminated PA I38 and PA T38 with high sensitivity.

**Conclusions:**

Our assays should be considered a powerful tool for monitoring the emergence of baloxavir‐resistant influenza viruses.

## 1 INTRODUCTION

In Japan, the novel antiviral drug baloxavir marboxil (S‐033188) was approved in February 2018 for the treatment of influenza A and B virus infection in patients 12 years or older and in children younger than 12 years weighing 10 kg or more; it became available in March 2018. The hydrolyzed active form of baloxavir marboxil (baloxavir acid; S‐033447) inhibits the cap‐dependent endonuclease of influenza A and B viruses.^1^ In vitro studies have revealed that an I38T substitution in the polymerase acidic subunit (PA) is associated with reduced susceptibility of influenza A(H1N1)pdm09, A(H3N2), and B viruses to baloxavir.^2^, ^3^ Furthermore, in a phase II clinical trial,^3^ PA I38T and I38F substitutions emerged following exposure to baloxavir marboxil in four (3.6%) of 112 influenza A(H1N1)pdm09 viruses for which PA sequences were available. Moreover, in a phase III clinical trial, PA I38T and I38M substitutions were detected after exposure to the drug in 9.7% of 370 influenza A(H3N2) viruses.^4^ Patients infected with mutant influenza viruses carrying PA I38T or I38F exhibited prolonged viral shedding, and the median time to alleviation of symptoms was longer in baloxavir recipients infected with viruses with PA I38T or I38M substitutions than in those without substitutions.^3^, ^4^ In a pediatric study, PA I38T and I38M substitutions emerged in 18 (23.4%) of 77 influenza A(H3N2) viruses.^3^

In Japan, two approaches have been developed to monitor the emergence of baloxavir‐resistant influenza viruses since the 2017/2018 influenza season: the focus reduction assay, which assesses baloxavir susceptibility, and sequence analysis by next‐generation sequencing (NGS), which detects amino acid substitutions at amino acid residue 38 in PA.^5^ Using NGS, in December 2018, we detected the PA I38T substitution in two clinical strains isolated from two patients 3 days after baloxavir administration.^6^ As of January 2019, PA I38T substitution was found in 11 cases infected with influenza A(H3N2) viruses, including one patient untreated with baloxavir.^7^ Although monitoring by NGS in Japan works well, this method requires skill and is labor‐intensive, and the results can take 3 days to acquire.

RNase H2‐dependent PCR (rhPCR) was recently established to enable the performance of PCR using blocked primers containing a single ribonucleotide residue that are activated by cleavage with a thermophilic archaeal RNase H2 enzyme from *Pyrococcus abyssi*.^8^ rhPCR was found to be more sensitive than standard allele‐specific PCR when applied to the detection of single‐nucleotide polymorphisms (SNPs).^8^ In this study, for the rapid and simple monitoring of influenza viruses possessing the I38T substitution in PA in a laboratory, three rhPCR‐based assays were developed for influenza A(H1N1)pdm09, A(H3N2), and B viruses, referred to as A/H1pdm PA_I38T rhPCR, A/H3 PA_I38T rhPCR, and B PA_I38T rhPCR, respectively. We evaluated these assays using cDNAs synthesized from in vitro‐transcribed RNA controls, RNAs purified from isolated viruses and clinical specimens. The assays developed in this study could be helpful for monitoring the emergence of baloxavir‐resistant influenza viruses.

## 2 MATERIALS AND METHODS

### 2.1 Primer design

Three rhAmp SNP Assays (primer sets) for A/H1pdm PA_I38T rhPCR, A/H3 PA_I38T rhPCR, and B PA_I38T rhPCR were designed on the basis of the consensus *PA* gene sequence of A(H1N1)pdm09, A(H3N2), and B viruses isolated in the 2017/2018 influenza season using the rhAmp^®^ Genotyping Design Tool (Integrated DNA Technologies) (Table 1). Each rhAmp SNP Assay consisted of one gene‐specific primer and two allele‐specific primers. One allele‐specific primer was designed to detect a T at nucleotide 113 (encoding an Ile at amino acid residue 38: PA I38) and was detected with a Yakima Yellow–labeled universal probe, and the other was for detection of a C at nucleotide 113 (encoding a Thr at amino acid residue 38: PA T38) and was detected by a FAM‐labeled universal probe.

**Table 1:** Primers for rhAmp SNP Assays

Name	Sequence (5′‐3′)	Position^a^
Primers for A/H1pdm PA_I38T rhPCR (Assay ID: CD.GT.BSFN7032.1)		
A/H1pdm_PA rhAmp‐FY^b^	UFP1/AAACTTCCAAATGTGTGCAAArUTGCA	108‐133
A/H1pdm_PA rhAmp‐FF^c^	UFP2/AAACTTCCAAATGTGTGCAAGrUTGCA	108‐133
A/H1pdm_PA rhAmp‐R^d^	GCTGTGCGACAATGCTTCAATrCCAAT	12‐35
Primers for A/H3 PA_I38T rhPCR (Assay ID: CD.GT.BGPR9916.4)		
A/H3_PA rhAmp‐FY^b^	UFP1/CACCTCCAAGTGAGTGCATArUTGCT	108‐132
A/H3_PA rhAmp‐FF^c^	UFP2/ACCTCCAAGTGAGTGCATGrUTGCT	108‐131
A/H3_PA rhAmp‐R^d^	GCTGTGCGACAATGCTTCAACrCCGAT	12‐35
Primers for B PA_I38T rhPCR (Assay ID: CD.GT.BGDB2770.1)		
B_PA rhAmp‐FY^b^	UFP1/CCAGCAATGCTATTCAACATrCTGTG	94‐118
B_PA rhAmp‐FF^c^	UFP2/CCAGCAATGCTATTCAACACrCTGTG	94‐118
B_PA rhAmp‐R^d^	GCCTAATGCTGTATATGCTTTTCCTTrCTTCG	165‐193

a Nucleotide positions of *PA* genes are based on cRNA sequences obtained from the GISAID database. Isolate ID numbers of A/Niigata‐C/66/2017 (A/H1pdm), A/Kanagawa/AC1731/2018 (A/H3), and B/YOKOHAMA/62/2017 are EPI_ISL_305545, EPI_ISL_311974, and EPI_ISL_286767, respectively.

b Allele‐specific primer contained universal forward primer 1 (UFP1), detected by Yakima Yellow–labeled universal probe for detection of I38.

c Allele‐specific primer contained universal forward primer 2 (UFP2), detected by FAM‐labeled universal probe for detection of T38.

d Gene‐specific primer.

### 2.2 Viruses and clinical specimens

Influenza viruses were isolated using Madin‐Darby canine kidney (MDCK) cells, MDCK‐SIAT1 cells,^9^ AX‐4 cells,^10^ or Caco‐2 cells, and typing and subtyping of clinical isolates were performed by hemagglutination inhibition (HI) test or real‐time RT‐PCR.^11^, ^12^ All clinical strains used in this study were collected as part of the work of the National Epidemiological Surveillance of Infectious Diseases in Japan.

Nasal or pharyngeal swabs collected from suspected and contact cases of influenza suspended in virus transport medium were obtained from Eiju General Hospital, Tokyo, Japan, between September 2018 and January 2019. The study protocol was approved by the Ethics Committee at Eiju General Hospital (No. 2015‐34) and the National Institute of Infectious Diseases (No. 1016), and the study was performed in compliance with the Declaration of Helsinki.

A total of 184, 91, and 236 clinical strains of influenza A(H1N1)pdm09, A(H3N2), and B viruses, respectively, isolated in the 2017/2018 influenza season were used in this study and are listed in Tables S1‐S3. Twelve clinical strains isolated in the 2018/2019 influenza season and the 11 clinical specimens used in this study are listed in Table 3.

### 2.3 RNA purification

RNA was purified from clinical isolates and clinical specimens using the QIAcube and QIAamp Viral RNA Mini QIAcube Kit (Qiagen) according to the manufacturer's protocol.

### 2.4 Preparation of RNA controls

*PA* genes with a T at nucleotide 113 were amplified from A/NIIGATA‐C/66/2017 (A/H1pdm), A/KANAGAWA/AC1731/2018 (A/H3), and B/YOKOHAMA/62/2017 (B) (GISAID isolate IDs: EPI_ISL_305545, EPI_ISL_311974, and EPI_ISL_286767) by RT‐PCR with the primer pairs listed in Table S4 using the SuperScript III One‐Step RT‐PCR System with Platinum Taq DNA Polymerase (Thermo Fisher Scientific, Waltham, MA, USA). The resulting PCR product of A/H3 was cloned using a TOPO TA Cloning Kit for Sequencing (Thermo Fisher Scientific), and those of A/H1pdm and B were cloned into the pHH21 vector.^13^ A mutation from T to C at nucleotide 113 was introduced into each gene using the primer pairs listed in Table S4 and the QuikChange Lightning Site‐Directed Mutagenesis Kit (Agilent Technologies). From the resulting six plasmids, *PA* genes possessing T or C at nucleotide 113 were amplified with the primer pairs listed in Table S4 by PCR using Phusion High‐Fidelity DNA Polymerase (New England BioLabs). From the resulting PCR products containing the T7 promoter, in vitro‐transcribed *PA* RNAs were synthesized as described previously.^14^

### 2.5 Reverse transcription

Reverse transcription was performed to synthesize cDNA from RNA controls and RNA purified from clinical isolates and clinical specimens with the PrimeScript™ RT Reagent Kit (Perfect Real Time) (Takara). Each 10‐µL reaction mix contained 2 µL of 5× PrimeScript buffer, 0.5 µL of PrimeScript RT Enzyme Mix, 0.5 µL of primer (2 µmol/L), 2 µL of water, and 5 µL of template RNA. cDNA was synthesized at 42°C for 15 minutes and 85°C for 5 seconds using a C1000™ Thermal Cycler (Bio‐Rad). Primers for reverse transcription of control RNAs were as follows: A_PA_F (ATGGAAGAYTTTGTGCGA) for influenza A virus RNA controls and B_PA_F (ATGGATACYTTTATTACAAGAAAC) for influenza B virus RNA controls. Primers for reverse transcription of clinical specimens and clinical isolates were as follows: Uni12 (AGCAAAAGCAGG)^15^ for influenza A viruses and Uni9 (AGCAGAAGC)^16^ for influenza B viruses.

### 2.6 rhPCR

rhPCR of cDNA was performed using the rhAmp^®^ SNP Genotyping System (Integrated DNA Technologies). Each 10‐µL assay contained 5 µL of rhAmp Genotyping Master Mix, 0.25 µL of rhAmp Reporter Mix, 0.5 µL of rhAmp SNP Assay (Table 1), 2.25 µL of water, and 2 µL of template cDNA. Cycling was performed as follows: enzyme activation for 10 minutes at 95°C, followed by 40 cycles of amplification (denaturation at 95°C for 10 seconds, annealing at 60°C for 30 seconds, and extension at 68°C for 20 seconds) using a LightCycler^®^ 480 (Roche). Fluorescent signals were collected during the extension step, and amplification data and endpoint data were analyzed using Light Cycler^®^ 480 SW1.5 software according to the manufacturer's instructions.

### 2.7 Deep sequencing

The *PA* gene was amplified from viral RNA using the SuperScript III One‐step RT‐PCR system with Platinum Taq (Thermo Fisher Scientific) and universal primers.^17^ A DNA library was prepared from RT‐PCR products using a QIAseq FX DNA Library Kit (Qiagen), followed by purification by Agencourt AMPure XP (Beckman Coulter). The library was sequenced with MiSeq Reagent Kits v3 and the MiSeq system (Illumina). Sequence reads were aligned to reference sequences using CLC Genomics Workbench 11 (Qiagen). A minor allele frequency threshold of 10% was used for the detection of SNPs. All sequences are available from the EpiFlu database of the Global Initiative on Sharing All Influenza Data (GISAID) (Table 3).

### 2.8 Statistical analysis

Probit analysis was performed using statplus software (AnalystSoft Inc).

## 3 RESULTS

### 3.1 Development of rhPCR assays

Three rhPCR‐based assays, A/H1pdm PA_I38T rhPCR, A/H3 PA_I38T rhPCR, and B PA_I38T rhPCR, were developed to discriminate between influenza A(H1N1)pdm09, A(H3N2), and B viruses carrying PA I38 and PA T38, respectively (Table 1). These assays were developed for analysis of clinical strains and/or viruses contained in clinical specimens that had already been typed and/or subtyped using some other method.

**Figure 1:**
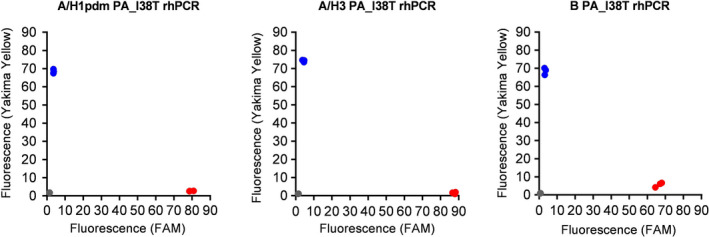
Endpoint fluorescence plots of A/H1pdm PA_I38T rhPCR, A/H3 PA_I38T rhPCR, and B PA_I38T rhPCR. Relative I38 (Yakima Yellow) and T38 (FAM) fluorescence intensities are plotted on the *y*‐axis and *x*‐axis, respectively. For each assay, 1000 copies/reaction of template cDNA were synthesized by reverse transcription from each RNA control. RNA controls included the PA gene segments of A/Niigata‐C/66/2017(A/H1pdm), A/Kanagawa/AC1731/2018 (A/H3), and B/YOKOHAMA/62/2017 (B) carrying I38 (blue circles) or T38 (red circles). The plot was generated using the results obtained from three replicates for each assay. Gray circles represent the results of negative controls (water)

The specificity of each assay for discriminating between viruses possessing PA I38 and PA T38 was evaluated using 1000 copies/reaction of cDNA synthesized from each RNA control in three replicates. As shown in Figure 1, each assay accurately discriminated between controls carrying PA I38 and PA T38.

The detection limit of each assay was determined by a Probit analysis using cDNA synthesized from serially diluted control RNAs, with six replicates for each assay (Table 2). The detection limits of A/H1pdm PA_I38T rhPCR for PA I38 and PA T38 were 36.8 and 34.9 copies/reaction, respectively. Those of A/H3 PA_I38T rhPCR for PA I38 and PA T38 were 29.6 and 32.5 copies/reaction, respectively, and those of B PA_I38T rhPCR for PA I38 and PA T38 were 29.3 and 33.9 copies/reaction, respectively.

**Table 2:** Detection limits of rhPCR assays

Template cDNA concentration^a^ (copies/reaction)	No. of positive replicates/No. of tests for each assay					
	A/H1pdm PA_I38T^‡^		A/H3 PA_I38T^§^		B PA_I38T^¶^	
	I38	T38	I38	T38	I38	T38
50	6/6	6/6	6/6	6/6	6/6	6/6
25	6/6	5/6	6/6	6/6	6/6	6/6
10	0/6	3/6	6/6	4/6	5/6	3/6
5	0/6	1/6	3/6	0/6	3/6	0/6
1	0/6	0/6	0/6	0/6	0/6	0/6
0	0/6	0/6	0/6	0/6	0/6	0/6

Note

The template cDNA for each assay was synthesized by reverse transcription from each RNA control, namely the *PA* gene segments of ^‡^A/Niigata‐C/66/2017 (A/H1pdm), ^§^A/Kanagawa/AC1731/2018 (A/H3), and ^¶^B/YOKOHAMA/62/2017 (B) carrying I38 or T38.

a Concentrations of cDNA were calculated from concentrations of RNA controls with a reverse transcription rate of 100%.

**Figure 2:**
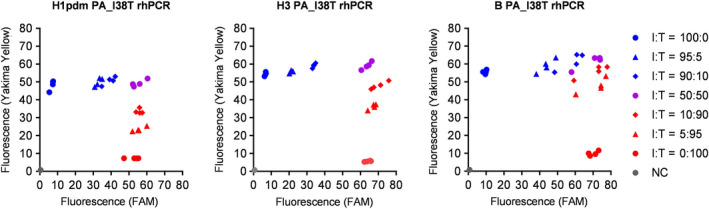
Endpoint fluorescence plot of A/H1pdm PA_I38T rhPCR, A/H3 PA_I38T rhPCR, and B PA_I38T rhPCR. Relative I38 (Yakima Yellow) and T38 (FAM) fluorescence are plotted on the *y*‐axis and *x*‐axis, respectively. The template cDNA for each assay was synthesized by reverse transcription from a mixture of each RNA control: a ratio of I38 to T38 of 100:0 (blue circle), 95:5 (blue triangle), 90:10 (blue square), 50:50 (purple circle), 10:90 (red square), 5:95 (red triangle), and 0:100 (red circle). Two hundred copies/reaction of cDNA were used. The plot was generated using the results obtained from each assay performed in four replicates. Gray circles represent the results of the negative control (water)

By using mixtures of PA I38 and PA T38 RNA controls each with a ratio of I38 to T38 of 100:0, 95:5, 90:10, 50:50, 10:90, 5:95, and 0:100, a lower percentage of limit of detection was tested for each assay. A total of 200 copies/reaction of cDNA synthesized from each mixture of RNA controls were used in four replicates. As shown in Figure 2, each assay was able to detect at least 5% of I38 or T38 in the mixture.

### 3.2 Validation of rhPCR assays performed using clinical strains and clinical specimens

Among strains isolated in the 2017/2018 influenza season, 184 influenza A(H1N1)pdm09 strains, 91 influenza A(H3N2) strains, and 236 influenza B strains (Tables S1‐S3) were tested by A/H1pdm PA_I38T rhPCR, A/H3 PA_I38T rhPCR, and B PA_I38T rhPCR, respectively. All strains were determined to carry PA I38 (data not shown).

**Figure 3:**
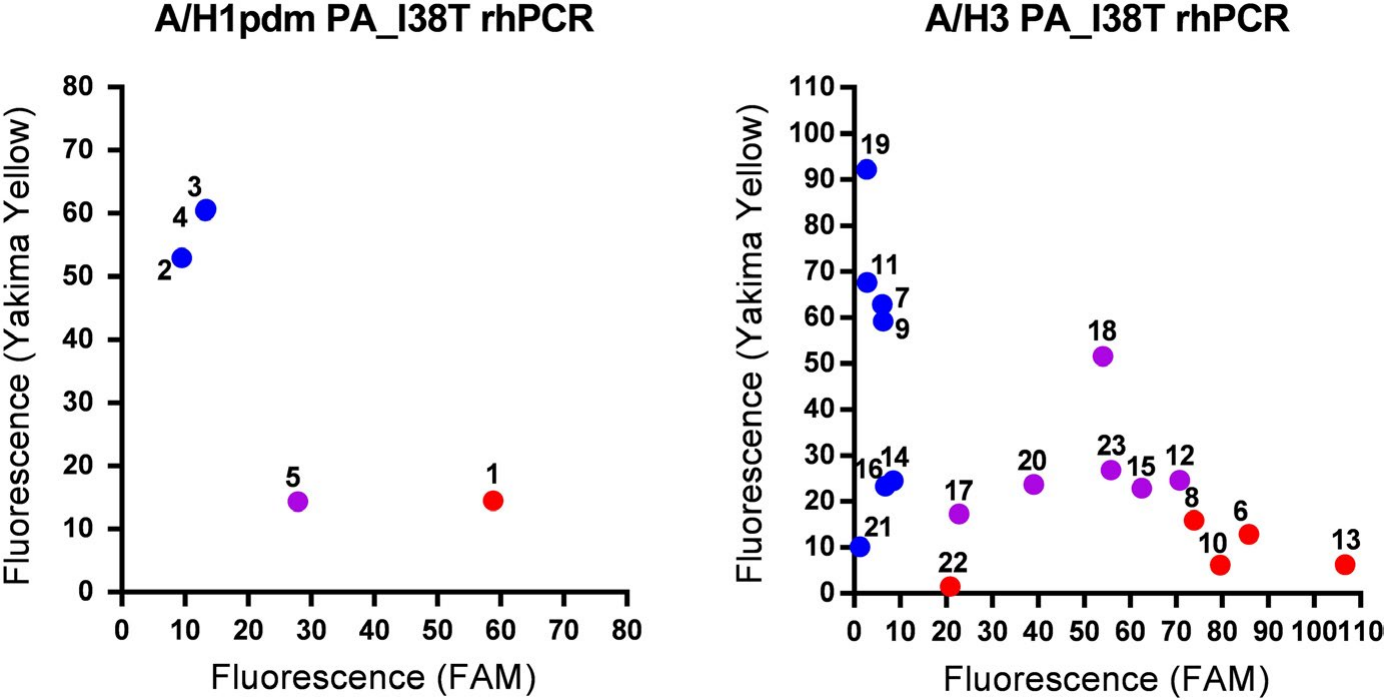
Endpoint fluorescence plots of A/H1pdm PA_I38T rhPCR and A/H3 PA_I38T rhPCR using cDNA synthesized from RNAs of clinical strains and clinical specimens. Relative I38 (Yakima Yellow) and T38 (FAM) fluorescence intensities are plotted on the *y*‐axis and *x*‐axis, respectively. Influenza viruses discriminated as I38, T38, or a mixture of I38 and T38 are represented as blue, red, or purple circles, respectively. Numbers in the figure indicate virus numbers listed in Table 3

Four influenza A(H1N1)pdm09 strains isolated in the 2018/2019 influenza season and one clinical specimen positive for A(H1N1)pdm09 were tested by both A/H1pdm PA_I38T rhPCR and NGS. Similarly, eight influenza A(H3N2) strains and ten clinical specimens positive for A(H3N2) were tested by A/H3 PA_I38T rhPCR and NGS. As shown in Figure 3 and Table 3, three of the influenza A(H1N1)pdm09 strains were determined to carry PA I38 by rhPCR and NGS. The remaining strain was found to carry PA T38 by rhPCR and appeared as a mixture of PA T38 and F38 according to NGS. The influenza A(H1N1)pdm09 virus derived from the clinical specimen was determined to be a mixture of PA I38 and T38 by rhPCR and NGS. Three, four, and one influenza A(H3N2) strains were determined to carry PA I38, T38, or a mixture of I38 and T38 according to rhPCR and NGS. Four, one, and five influenza A(H3N2) viruses from clinical specimens were found to carry PA I38, T38, or a mixture of I38 and T38 by rhPCR. In contrast, two, one, one, one, and five of these viruses were determined to carry PA I38; T38; M38; a mixture of I38, T38, and M38; or a mixture of I38 and T38 according to NGS.

**Table 3:** Clinical strains and clinical specimens isolated and collected in the 2018/2019 influenza season and tested by rhPCR assays

No.	Name	GISAID ID	Type/subtype	Amino acid at PA 38	
				rhPCR	NGS
1	A/KANAGAWA/88/2018	EPI_ISL_337454	A/H1pdm (Clinical strain)	T	T: 59% F: 41%
2	A/YOKOHAMA/123/2018	EPI_ISL_333604	A/H1pdm (Clinical strain)	I	I: 100%
3	A/YOKOHAMA/124/2018	EPI_ISL_333605	A/H1pdm (Clinical strain)	I	I: 100%
4	A/YOKOHAMA/125/2018	EPI_ISL_333606	A/H1pdm (Clinical strain)	I	I: 100%
5	A/KANAGAWA/IC1890/2019	EPI_ISL_345217	A/H1pdm (Clinical specimen)	Mix	T: 72% I: 28%
6	A/YOKOHAMA/133/2018	EPI_ISL_332908	A/H3 (Clinical strains)	T	T: 100%^†^
7	A/YOKOHAMA/134/2018	EPI_ISL_332909	A/H3 (Clinical strain)	I	I: 100%^†^
8	A/YOKOHAMA/135/2018	EPI_ISL_332910	A/H3 (Clinical strain)	T	T: 100%^†^
9	A/YOKOHAMA/136/2018	EPI_ISL_332911	A/H3 (Clinical strain)	I	I: 100%^†^
10	A/YOKOHAMA/56/2019	EPI_ISL_340695	A/H3 (Clinical strain)	T	T: 100%^‡^
11	A/YOKOHAMA/60/2019	EPI_ISL_340697	A/H3 (Clinical strain)	I	I: 100%
12	A/YOKOHAMA/61/2019	EPI_ISL_340699	A/H3 (Clinical strain)	Mix	T: 88% I: 12%^‡^
13	A/MIE/41/2018	EPI_ISL_346647	A/H3 (Clinical strain)	T	T: 100%
14	A/KANAGAWA/IC1807/2018	EPI_ISL_340686	A/H3 (Clinical specimen)	I	I: 90% T: 10%^‡^
15	A/KANAGAWA/AC1817/2018	EPI_ISL_337452	A/H3 (Clinical specimen)	Mix	T: 59% I: 21% M: 20%
16	A/KANAGAWA/IC1817/2019	EPI_ISL_340691	A/H3 (Clinical specimen)	I	I: 100%
17	A/KANAGAWA/IC1827/2019	EPI_ISL_337459	A/H3 (Clinical specimen)	Mix	I: 84% T: 16%^‡^
18	A/KANAGAWA/AC1829/2019	EPI_ISL_337458	A/H3 (Clinical specimen)	Mix	I: 82% T: 18%^‡^
19	A/KANAGAWA/IC18154/2019	EPI_ISL_345223	A/H3 (Clinical specimen)	I	I: 100%
20	A/KANAGAWA/IC1869/2019	EPI_ISL_345211	A/H3 (Clinical specimen)	Mix	I: 84% T: 16%
21	A/KANAGAWA/IC1870/2019	EPI_ISL_345212	A/H3 (Clinical specimen)	I	M: 100%
22	A/KANAGAWA/IC1894/2019	EPI_ISL_345218	A/H3 (Clinical specimen)	T	T: 100%
23	A/KANAGAWA/IC18102/2019	EPI_ISL_345219	A/H3 (Clinical specimen)	Mix	T: 89% I: 11%

Note

NGS data from references ^†^ 6 and ^‡^ 7.

## 4 DISCUSSION

Regardless of the type and subtype of influenza virus, all clinical strains isolated in the 2017/2018 influenza season were determined to carry PA I38 by A/H1pdm PA_I38T rhPCR, A/H3 PA_I38T rhPCR, and B PA_I38T rhPCR. These results are in agreement with previous reports that PA I38 is highly conserved among influenza A and B viruses,^3^ that the I38T substitution in PA was not detected in seasonal influenza strains isolated in the 2017/2018 influenza season in Japan,^18^ and that influenza viruses circulating in the Asia‐Pacific region between 2012 and 2018 were susceptible to baloxavir.^19^

Five and 18 influenza A(H1N1)pdm09 and A(H3N2) clinical strains or viruses contained in clinical specimens isolated or collected in the 2018/2019 influenza season were tested by A/H1pdm PA_I38T rhPCR and A/H3 PA_I38T rhPCR, respectively. The results of the rhPCR assays for discriminating between PA I38 and PA T38 corresponded with the results of NGS except for A/KANAGAWA/IC1807/2018. According to A/H3 PA_I38T rhPCR, A/KANAGAWA/IC1807/2018 was determined to carry PA I38, whereas a mixture of PA I38 and T38 was detected by NGS. Detailed NGS analysis revealed that the percentage of PA T38 contained in the clinical specimen of A/KANAGAWA/IC1807/2018 was 10%. By using in vitro‐transcribed RNA controls, A/H3 PA_I38T rhPCR was able to detect at least 5% of T38 in the mixture, whereas it failed to detect 10% of PA T38 in A/KANAGAWA/IC1807/2018. The discrepancy between the results from RNA controls and from clinical specimens may be caused by the difference in RNA purity between in vitro‐transcribed RNA and RNA purified from a clinical specimen. Alternatively, the discrepancy between the results from the rhPCR assay and from NGS may be caused by the different the respective experimental procedures after the RNA purification step. Further evaluation with clinical specimens containing a mixture of PA I38 and T38 from future influenza seasons would be required to explain the discrepancy.

In two clinical strains, A/KANAGAWA/AC1817/2018 and A/KANAGAWA/IC1870/2019, NGS detected PA M38. A/H3 PA_I38T rhPCR was designed to discriminate between PA I38 (T at nucleotide 113) and T38 (C at nucleotide 113), not to identify M38, which is also encoded by a T at nucleotide 113; thus, rhPCR reported the amino acid as PA I38. In a clinical strain, A/KANAGAWA/88/2018, NGS detected F38. Similarly, A/H1pdm PA_I38T rhPCR was not designed to identify F38, which is encoded by a T at nucleotides 112 and 113. In this case, rhPCR only detected PA T38 in A/KANAGAWA/88/2018 and failed to detect F38.

In previous analyses involving the focus reduction assay, comparison of median IC_50_ values indicated that influenza A(H3N2) clinical strains carrying PA T38 in Japan exhibited >100‐fold reduced susceptibilities to baloxavir compared to those of influenza A(H3N2) clinical strains carrying PA I38.^5^, ^6^, ^7^ In contrast, influenza viruses carrying the PA I38M or PA I38F substitutions exhibited only moderate reductions in susceptibility to baloxavir.^3^, ^4^, ^20^ As of July 2019, five A(H1N1)pdm09 and 30 A(H3N2) viruses in Japan had been found to carry a mutation at PA amino acid residue 38 by NGS.^18^ Of these, two A(H1N1)pdm09 viruses carried PA I38F or I38S substitutions, and two A(H3N2) viruses carried PA I38M substitution. However, the remaining A(H1N1)pdm09 and A(H3N2) viruses were found to carry PA T38 or a mixture including PA T38. It was also reported that PA T38 was the most common substitution emerging during baloxavir treatment in Japan and in the United States.^21^, ^22^, ^23^ These results suggest that our developed rhPCR assays should be able to detect the majority of PA I38 mutant viruses.

The rhPCR assays developed in this study can rapidly detect already typed and/or subtyped influenza viruses carrying the I38T substitution in PA with high sensitivity and specificity within 3‐4 hours, and can be performed on many samples at a time. Based on these results, rhPCR assays should be considered a useful tool for the screening of the PA I38T substitution and monitoring of the emergence of baloxavir‐resistant influenza viruses.

## References

[1] Koszalka P , Tilmanis D , Hurt AC . Influenza antivirals currently in late‐phase clinical trial. Influenza Other Respir Viruses. 2017;11(3):240‐246. 28146320 10.1111/irv.12446 PMC5410715

[2] Noshi T , Kitano M , Taniguchi K , et al. In vitro characterization of baloxavir acid, a first‐in‐class cap‐dependent endonuclease inhibitor of the influenza virus polymerase PA subunit. Antiviral Res. 2018;160:109‐117. 30316915 10.1016/j.antiviral.2018.10.008

[3] Omoto S , Speranzini V , Hashimoto T , et al. Characterization of influenza virus variants induced by treatment with the endonuclease inhibitor baloxavir marboxil. Sci Rep. 2018;8(1):9633. 29941893 10.1038/s41598-018-27890-4 PMC6018108

[4] Hayden FG , Sugaya N , Hirotsu N , et al. Baloxavir marboxil for uncomplicated influenza in adults and adolescents. N Engl J Med. 2018;379(10):913‐923. 30184455 10.1056/NEJMoa1716197

[5] Takashita E , Morita H , Ogawa R , et al. Susceptibility of influenza viruses to the novel cap‐dependent endonuclease inhibitor baloxavir marboxil. Front Microbiol. 2018;9:3026. 30574137 10.3389/fmicb.2018.03026 PMC6291754

[6] Takashita E , Kawakami C , Morita H ,, et al. Detection of influenza A(H3N2) viruses exhibiting reduced susceptibility to the novel cap‐dependent endonuclease inhibitor baloxavir in Japan, December 2018. Euro Surveill. 2019;24(3). 10.2807/1560-7917.ES.2019.24.3.1800698 PMC6344841 30670142

[7] Takashita E , Kawakami C , Ogawa R ,, et al. Influenza A(H3N2) virus exhibiting reduced susceptibility to baloxavir due to a polymerase acidic subunit I38T substitution detected from a hospitalised child without prior baloxavir treatment, Japan, January 2019. Euro Surveill. 2019;24(12). 10.2807/1560-7917.ES.2019.24.12.1900170 PMC6440584 30914078

[8] Dobosy JR , Rose SD , Beltz KR , et al. RNase H‐dependent PCR (rhPCR): improved specificity and single nucleotide polymorphism detection using blocked cleavable primers. BMC Biotechnol. 2011;11:80. 21831278 10.1186/1472-6750-11-80 PMC3224242

[9] Matrosovich M , Matrosovich T , Carr J , Roberts NA , Klenk HD . Overexpression of the alpha‐2,6‐sialyltransferase in MDCK cells increases influenza virus sensitivity to neuraminidase inhibitors. J Virol. 2003;77(15):8418‐8425. 12857911 10.1128/JVI.77.15.8418-8425.2003 PMC165236

[10] Hatakeyama S , Sakai‐Tagawa Y , Kiso M , et al. Enhanced expression of an alpha2,6‐linked sialic acid on MDCK cells improves isolation of human influenza viruses and evaluation of their sensitivity to a neuraminidase inhibitor. J Clin Microbiol. 2005;43(8):4139‐4146. 16081961 10.1128/JCM.43.8.4139-4146.2005 PMC1233980

[11] Nakauchi M , Yasui Y , Miyoshi T , et al. One‐step real‐time reverse transcription‐PCR assays for detecting and subtyping pandemic influenza A/H1N1 2009, seasonal influenza A/H1N1, and seasonal influenza A/H3N2 viruses. J Virol Methods. 2011;171(1):156‐162. 21029748 10.1016/j.jviromet.2010.10.018 PMC7173154

[12] Nakauchi M , Takayama I , Takahashi H , et al. Real‐time RT‐PCR assays for discriminating influenza B virus Yamagata and Victoria lineages. J Virol Methods. 2014;205:110‐115. 24797457 10.1016/j.jviromet.2014.04.016 PMC7172331

[13] Neumann G , Watanabe T , Ito H , et al. Generation of influenza A viruses entirely from cloned cDNAs. Proc Natl Acad Sci USA. 1999;96(16):9345‐9350. 10430945 10.1073/pnas.96.16.9345 PMC17785

[14] Nakauchi M , Takayama I , Takahashi H , et al. Development of real‐time fluorescent reverse transcription loop‐mediated isothermal amplification assays for rhinovirus detection. J Med Virol. 2019;91(7):1232‐1238. 30735248 10.1002/jmv.25427 PMC7166982

[15] Hoffmann E , Stech J , Guan Y , Webster RG , Perez DR . Universal primer set for the full‐length amplification of all influenza A viruses. Arch Virol. 2001;146(12):2275‐2289. 11811679 10.1007/s007050170002

[16] Fendrick AM , Monto AS , Nightengale B , Sarnes M . The economic burden of non‐influenza‐related viral respiratory tract infection in the United States. Arch Intern Med. 2003;163(4):487‐494. 12588210 10.1001/archinte.163.4.487

[17] Zhou B , Donnelly ME , Scholes DT , et al. Single‐reaction genomic amplification accelerates sequencing and vaccine production for classical and Swine origin human influenza a viruses. J Virol. 2009;83(19):10309‐10313. 19605485 10.1128/JVI.01109-09 PMC2748056

[18] National Institute of Infectious Diseases . Antiviral resistance surveillance in Japan 2019. https://www.niid.go.jp/niid/en/influ-resist-e.html. Accessed July 30, 2019.

[19] Koszalka P , Tilmanis D , Roe M , Vijaykrishna D , Hurt AC . Baloxavir marboxil susceptibility of influenza viruses from the Asia‐Pacific, 2012–2018. Antiviral Res. 2019;164:91‐96. 30771405 10.1016/j.antiviral.2019.02.007

[20] Gubareva LV , Mishin VP , Patel MC , et al. Assessing baloxavir susceptibility of influenza viruses circulating in the United States during the 2016/17 and 2017/18 seasons. Euro Surveill. 2019;24(3). 10.2807/1560-7917.ES.2019.24.3.1800666 PMC6344838 30670144

[21] Uehara T , Hayden FG , Kawaguchi K , et al. Treatment‐emergent influenza variant viruses with reduced baloxavir susceptibility: impact on clinical and virologic outcomes in uncomplicated influenza. J Infect Dis. 2019;221(3):346‐355. 10.1093/infdis/jiz244 31309975

[22] Hirotsu N , Sakaguchi H , Sato C , et al. Baloxavir marboxil in Japanese pediatric patients with influenza: safety and clinical and virologic outcomes. Clin Infect Dis. 2019. In press. 10.1093/cid/ciz908 PMC7428393 31538644

[23] Gubareva LV , Fry AM . Baloxavir and treatment‐emergent resistance: public health insights and next steps. J Infect Dis. 2019;221(3):337‐339. 10.1093/infdis/jiz245 PMC9012239 31309982

