# Supplementary material for: Rapid detection of an I38T amino acid substitution in influenza polymerase acidic subunit associated with reduced susceptibility to baloxavir marboxil
Source: Influenza Other Respir Viruses. 2020 Feb 16;14(4):436–43. doi: 10.1111/irv.12728 (PMC7298299; doi:10.1111/irv.12728)
Supplement: Supplementary file 1 [file IRV-14-436-s001.docx]

Table S1. Influenza A(H1N1)pdm09 clinical strains isolated in 2017/2018 influenza season

| A/KANAGAWA/AC1703/2017 | A/KANAGAWA/AC1710/2017 | A/SENDAI/55/2017 |
| --- | --- | --- |
| A/SAPPORO/47/2017 | A/KANAGAWA/AC1711/2017 | A/SHIZUOKA-C/40/2017 |
| A/IWATE/24/2018 | A/KANAGAWA/IC1715/2017 | A/KANAGAWA/ZC1730/2018 |
| A/SAPPORO/48/2017 | A/TOKYO/17317/2017 | A/HIROSHIMA/5/2018 |
| A/KYOTO/86/2017 | A/KANAGAWA/AC1713/2018 | A/SHIMANE/111/2017 |
| A/KYOTO/91/2017 | A/AICHI/346/2017 | A/YOKOHAMA/149/2017 |
| A/KYOTO/99/2017 | A/NIIGATA/986/2017 | A/KANAGAWA/IC1741/2018 |
| A/SAPPORO/50/2017 | A/TOKYO/17318/2017 | A/CHIBA-C/3/2018 |
| A/KOBE/278/2017 | A/KAWASAKI/134/2017 | A/MIYAGI/80/2017 |
| A/KOBE/286/2017 | A/KAWASAKI/144/2017 | A/AICHI/341/2017 |
| A/KOBE/302/2017 | A/OSAKA/184/2017 | A/YAMAGATA/10/2018 |
| A/AOMORI/1/2017 | A/GIFU/61/2017 | A/YAMANASHI/17294/2017 |
| A/YOKOHAMA/155/2017 | A/OSAKA-C/10/2017 | A/KANAGAWA/AC1702/2017 |
| A/YOKOHAMA/159/2017 | A/CHIBA-C/53/2017 | A/SAGA/17/2018 |
| A/YOKOHAMA/168/2017 | A/IWATE/71/2017 | A/SHIMANE/1/2018 |
| A/KANAGAWA/83/2018 | A/SAITAMA/281/2017 | A/SAPPORO/73/2017 |
| A/TOKYO/18049/2018 | A/TOKYO/17433/2017 | A/SHIGA/4/2018 |
| A/KAWASAKI/135/2017 | A/KANAGAWA/234/2017 | A/KANAGAWA/ZC1732/2018 |
| A/KAWASAKI/137/2017 | A/NIIGATA-C/50/2017 | A/SHIZUOKA/59/2017 |
| A/KAWASAKI/142/2017 | A/WAKAYAMA/110/2017 | A/WAKAYAMA-C/134/2017 |
| A/YOKOHAMA/94/2018 | A/KANAGAWA/IC1723/2018 | A/GUNMA/165/2017 |
| A/KANAGAWA/ZC1736/2018 | A/KAWASAKI/133/2017 | A/YOKOHAMA/24/2018 |
| A/NAGANO-C/1/2018 | A/KANAGAWA/IC1724/2018 | A/KANAGAWA/ZC1702/2017 |
| A/TOKYO/18066/2018 | A/SAGA/269/2017 | A/KANAGAWA/AC1735/2018 |
| A/TOKYO/18069/2018 | A/YOKOSUKA/37/2017 | A/KANAGAWA/ZC1703/2017 |
| A/KANAGAWA/AC1705/2017 | A/MIE/48/2017 | A/SAITAMA-C/1/2018 |
| A/KANAGAWA/AC1706/2017 | A/SHIMANE/108/2017 | A/FUKUI/12/2017 |
| A/SAITAMA-C/22/2017 | A/ISHIKAWA/120/2017 | A/FUKUI/1/2018 |
| A/KANAGAWA/IC1704/2017 | A/HYOGO/1095/2017 | A/FUKUI/14/2017 |
| A/KANAGAWA/ZC1707/2017 | A/KANAGAWA/ZC1713/2018 | A/FUKUOKA-C/3/2018 |
| A/KANAGAWA/IC1705/2017 | A/KANAGAWA/AC1718/2018 | A/YAMANASHI/18067/2018 |
| A/NIIGATA/923/2017 | A/KANAGAWA/AC1720/2018 | A/HYOGO/1188/2018 |
| A/MIE/34/2017 | A/TOCHIGI/17153/2017 | A/SAGAMIHARA/45/2017 |
| A/YOKOHAMA/176/2017 | A/YOKOHAMA/3/2018 | A/OSAKA-C/1/2018 |
| A/KANAGAWA/IC1707/2017 | A/HIROSHIMA/77/2017 | A/KANAGAWA/ZC1704/2017 |
| A/SAITAMA/251/2017 | A/KANAGAWA/IC1729/2018 | A/KAWASAKI/159/2017 |
| A/KANAGAWA/ZC1708/2017 | A/KANAGAWA/IC1730/2018 | A/KAWASAKI/5/2018 |
| A/SAITAMA/257/2017 | A/KANAGAWA/IC1731/2018 | A/MIE/27/2017 |
| A/HIROSHIMA/83/2017 | A/KANAGAWA/IC1732/2018 | A/KANAGAWA/ZC1706/2017 |
| A/SAITAMA/252/2017 | A/KANAGAWA/ZC1715/2018 | A/FUKUOKA-C/18/2017 |
| A/MIYAZAKI/110/2017 | A/KANAGAWA/ZC1716/2018 | A/WAKAYAMA-C/60/2018 |
| A/YOKOHAMA/183/2017 | A/KANAGAWA/ZC1718/2018 | A/OKINAWA/7/2018 |
| A/YOKOHAMA/188/2017 | A/KANAGAWA/253/2017 | A/FUKUOKA-C/20/2017 |
| A/SAPPORO/56/2017 | A/HIROSHIMA-C/6/2017 | A/OKINAWA/11/2018 |
| A/SAPPORO/62/2017 | A/YAMAGATA/136/2017 | A/OKINAWA/27/2018 |
| A/SHIGA/32/2017 | A/YAMAGATA/142/2017 | A/SHIZUOKA/25/2018 |
| A/OITA/18/2017 | A/EHIME/4/2018 | A/YOKOHAMA/56/2018 |
| A/YOKOSUKA/28/2017 | A/KANAGAWA/ZC1720/2018 | A/NAGANO/2158/2018 |
| A/NAGANO/2639/2017 | A/KANAGAWA/ZC1721/2018 | A/SAITAMA/254/2017 |
| A/NAGANO/2660/2017 | A/KANAGAWA/AC1721/2018 | A/HIROSHIMA/33/2018 |
| A/NAGANO/2728/2017 | A/OSAKA/179/2017 | A/AICHI/119/2018 |
| A/KOBE/349/2017 | A/AOMORI/10/2017 | A/KOCHI/39/2017 |
| A/SAITAMA/268/2017 | A/AOMORI/12/2017 | A/KOCHI/10/2018 |
| A/CHIBA/35/2017 | A/AOMORI/12/2018 | A/KOCHI/17/2018 |
| A/EHIME/36/2017 | A/AOMORI/3/2018 | A/KANAGAWA/41/2018 |
| A/EHIME/43/2017 | A/AOMORI/9/2018 | A/KANAGAWA/59/2018 |
| A/OSAKA-C/12/2017 | A/KAGOSHIMA/74144/2017 | A/SAGAMIHARA/12/2018 |
| A/WAKAYAMA-C/109/2017 | A/TOKYO/17530/2017 | A/OSAKA/75/2018 |
| A/YOKOHAMA/209/2017 | A/AICHI/17/2018 | A/HIROSHIMA/40/2018 |
| A/KANAGAWA/IC1708/2017 | A/YAMAGUCHI/5/2018 | A/YOKOHAMA/81/2018 |
| A/HYOGO/1065/2017 | A/YAMANASHI/17210/2017 |  |
| A/KANAGAWA/IC1711/2017 | A/KANAGAWA/ZC1726/2018 |  |

Table S2. Influenza A(H3N2) clinical strains isolated in 2017/2018 influenza season

| A/KANAGAWA/215/2017 | A/KANAGAWA/AC1714/2018 | A/FUKUSHIMA/69/2017 |
| --- | --- | --- |
| A/YAMAGATA/164/2018 | A/SAITAMA-C/24/2017 | A/KANAGAWA/212/2017 |
| A/KAWASAKI/65/2018 | A/OSAKA/183/2017 | A/KANAGAWA/213/2017 |
| A/YOKOHAMA/160/2017 | A/TOKYO/17327/2017 | A/YOKOHAMA/14/2018 |
| A/YAMANASHI/17233/2017 | A/SHIMANE/112/2017 | A/AOMORI/7/2017 |
| A/KAWASAKI/136/2017 | A/KANAGAWA/IC1718/2018 | A/TOKYO/17351/2017 |
| A/KANAGAWA/AC1704/2017 | A/TOKYO/17330/2017 | A/GUNMA/140/2017 |
| A/KOBE/50/2018 | A/NIIGATA/888/2017 | A/AOMORI/6/2018 |
| A/FUKUSHIMA/33/2018 | A/CHIBA/30/2017 | A/KANAGAWA/AC1726/2018 |
| A/MIYAZAKI/72/2018 | A/MIYAZAKI/115/2017 | A/OSAKA/181/2017 |
| A/AICHI/199/2018 | A/KANAGAWA/IC1719/2018 | A/KANAGAWA/IC1737/2018 |
| A/TOKYO/17390/2017 | A/KANAGAWA/IC1721/2018 | A/KAGOSHIMA/74146/2017 |
| A/NIIGATA/922/2017 | A/SAITAMA/285/2017 | A/YOKOHAMA/147/2017 |
| A/YOKOSUKA/26/2017 | A/TOKYO/17427/2017 | A/HIROSHIMA/2/2018 |
| A/NIIGATA/836/2017 | A/WAKAYAMA/116/2017 | A/MIE/56/2017 |
| A/NIIGATA/847/2017 | A/SAGA/252/2017 | A/ISHIKAWA/118/2017 |
| A/KANAGAWA/223/2017 | A/MIE/53/2017 | A/IWATE/68/2017 |
| A/SAITAMA/261/2017 | A/GUNMA/136/2017 | A/NAGANO/2742/2017 |
| A/HIROSHIMA/80/2017 | A/YAMANASHI/17258/2017 | A/SHIZUOKA/60/2017 |
| A/TOCHIGI/17151/2017 | A/YAMAGUCHI/48/2017 | A/EHIME/18/2018 |
| A/YOKOHAMA/181/2017 | A/OSAKA/188/2017 | A/SAITAMA-C/2/2018 |
| A/YOKOHAMA/145/2017 | A/KYOTO-C/6/2017 | A/FUKUI/2/2018 |
| A/IWATE/69/2017 | A/MIE/30/2017 | A/AICHI/62/2018 |
| A/KOBE/348/2017 | A/MIE/32/2017 | A/YAMANASHI/18041/2018 |
| A/YAMAGATA/128/2017 | A/YOKOHAMA/232/2017 | A/SHIZUOKA-C/39/2017 |
| A/NIIGATA-C/51/2017 | A/GIFU/63/2017 | A/AICHI/343/2017 |
| A/OSAKA-C/79/2017 | A/MIE/3/2018 | A/NIIGATA/185/2018 |
| A/YOKOHAMA/179/2017 | A/KUMAMOTO/15/2017 | A/SAGAMIHARA/44/2017 |
| A/KANAGAWA/IC1713/2017 | A/AICHI/348/2017 | A/KYOTO-C/2/2018 |
| A/KANAGAWA/IC1714/2017 | A/SAKAI/25/2017 |  |
| A/KANAGAWA/IC1717/2018 | A/KANAGAWA/254/2017 |  |

Table S3. Influenza B clinical strains isolated in 2017/2018 influenza season

| B/KANAGAWA/ZC1701/2017 | B/KANAGAWA/ZC1728/2018 | B/OITA/12/2017 |
| --- | --- | --- |
| B/TOKYO/17329/2017 | B/KANAGAWA/IC1742/2018 | B/AICHI/73/2017 |
| B/KAWASAKI/74/2017 | B/NIIGATA/1066/2017 | B/GUNMA/137/2017 |
| B/YOKOHAMA/64/2017 | B/NIIGATA-C/1/2018 | B/SAGA/10030/2017 |
| B/KANAGAWA/IC1702/2017 | B/OSAKA/11/2018 | B/YOKOHAMA/3/2018 |
| B/SHIZUOKA/56/2017 | B/OSAKA/17/2018 | B/YOKOHAMA/93/2017 |
| B/SHIZUOKA/61/2017 | B/NAGANO/2059/2018 | B/GIFU/73/2017 |
| B/KANAGAWA/62/2017 | B/CHIBA-C/11/2018 | B/MIE/4/2018 |
| B/KANAGAWA/ZC1705/2017 | B/FUKUSHIMA/1/2018 | B/YOKOSUKA/30/2017 |
| B/HIROSHIMA/9/2017 | B/YOKOSUKA/3/2018 | B/TOKYO/17453/2017 |
| B/YOKOHAMA/71/2017 | B/FUKUI/9/2018 | B/FUKUSHIMA/36/2017 |
| B/KAWASAKI/75/2017 | B/NAGANO/2076/2018 | B/OSAKA/21/2017 |
| B/AICHI/62/2017 | B/NAGANO/2093/2018 | B/FUKUI/17/2017 |
| B/SAITAMA-C/21/2017 | B/NAGANO/2102/2018 | B/AKITA/5/2017 |
| B/NIIGATA/978/2017 | B/SAKAI/15/2018 | B/YAMAGATA/141/2017 |
| B/MIE/17/2017 | B/GUNMA/3/2018 | B/YOKOHAMA/11/2018 |
| B/MIE/20/2017 | B/AKITA/3/2018 | B/YOKOHAMA/13/2018 |
| B/CHIBA-C/51/2017 | B/AICHI/56/2018 | B/EHIME/3/2018 |
| B/KANAGAWA/IC1706/2017 | B/YAMANASHI/18055/2018 | B/EHIME/6/2017 |
| B/CHIBA/53/2017 | B/OSAKA/39/2018 | B/IWATE/37/2017 |
| B/HIROSHIMA/11/2017 | B/HYOGO/1152/2018 | B/SAGAMIHARA/50/2017 |
| B/SAPPORO/36/2017 | B/HYOGO/1196/2018 | B/NIIGATA-C/30/2017 |
| B/SHIGA/31/2017 | B/NIIGATA/173/2018 | B/SAITAMA-C/2/2018 |
| B/KOBE/347/2017 | B/NAGANO/2131/2018 | B/YAMAGUCHI/10/2018 |
| B/HYOGO/1076/2017 | B/YAMAGUCHI/15/2018 | B/MIYAGI/21/2017 |
| B/FUKUI/14/2017 | B/EHIME/25/2018 | B/KOBE/448/2018 |
| B/NIIGATA-C/24/2017 | B/TOKUSHIMA/5/2018 | B/NIIGATA-C/2/2018 |
| B/OSAKA-C/6/2017 | B/KAWASAKI/8/2018 | B/MIE/12/2018 |
| B/KANAGAWA/IC1709/2017 | B/KAWASAKI/60/2018 | B/SAPPORO/4/2018 |
| B/KANAGAWA/AC1712/2017 | B/KAGOSHIMA/84060/2018 | B/SAPPORO/41/2017 |
| B/KANAGAWA/IC1712/2017 | B/TOKUSHIMA/9/2018 | B/IWATE/6/2018 |
| B/KANAGAWA/AC1715/2018 | B/YAMANASHI/18163/2018 | B/NAGANO/2055/2018 |
| B/KAWASAKI/76/2017 | B/YOKOHAMA/87/2018 | B/SHIZUOKA/2/2018 |
| B/GIFU/56/2017 | B/OSAKA/77/2018 | B/AICHI/23/2018 |
| B/MIYAZAKI/113/2017 | B/KAGAWA/18095/2018 | B/NIIGATA/96/2018 |
| B/KANAGAWA/IC1703/2017 | B/SENDAI/15/2017 | B/NIIGATA/134/2018 |
| B/KANAGAWA/IC1720/2018 | B/AOMORI/49/2018 | B/SAPPORO/9/2018 |
| B/WAKAYAMA/109/2017 | B/AICHI/177/2018 | B/SAPPORO/11/2018 |
| B/KANAGAWA/IC1722/2018 | B/HIROSHIMA/91/2018 | B/GUNMA/10/2018 |
| B/MIE/25/2017 | B/OSAKA-C/31/2018 | B/FUKUOKA-C/5/2018 |
| B/NAGANO/2766/2017 | B/KANAGAWA/98/2018 | B/AICHI/64/2018 |
| B/NAGANO/2774/2017 | B/YAMAGATA/105/2018 | B/YOKOHAMA/58/2018 |
| B/OSAKA/13/2017 | B/YAMAGATA/150/2018 | B/YAMANASHI/18043/2018 |
| B/SHIZUOKA-C/10/2017 | B/KYOTO/20/2018 | B/HIROSHIMA/20/2018 |
| B/ISHIKAWA/121/2017 | B/NIIGATA-C/19/2018 | B/HYOGO/1207/2018 |
| B/NARA/6/2017 | B/TOKYO/17797/2018 | B/NIIGATA/235/2018 |
| B/KANAGAWA/AC1716/2018 | B/SAPPORO/38/2018 | B/CHIBA-C/38/2018 |
| B/KANAGAWA/IC1727/2018 | B/NIIGATA/440/2018 | B/MIYAGI/19/2018 |
| B/KANAGAWA/ZC1714/2018 | B/TOYAMA/19/2018 | B/NAGASAKI/643/2018 |
| B/KANAGAWA/AC1719/2018 | B/YAMAGUCHI/25/2018 | B/SHIMANE/25/2018 |
| B/GIFU/65/2017 | B/TOKYO/18009/2018 | B/SHIMANE/31/2018 |
| B/GIFU/70/2017 | B/KUMAMOTO-C/4/2018 | B/AICHI/82/2018 |
| B/KANAGAWA/IC1733/2018 | B/NAGANO-C/44/2018 | B/KAWASAKI/83/2017 |
| B/KANAGAWA/ZC1717/2018 | B/AICHI/180/2018 | B/FUKUI/30/2018 |
| B/KANAGAWA/ZC1719/2018 | B/AMAGASAKI/1187/2018 | B/WAKAYAMA/60/2018 |
| B/HIROSHIMA-C/1/2017 | B/MIYAGI/43/2018 | B/YAMAGATA/84/2018 |
| B/FUKUI/19/2017 | B/OKAYAMA/15/2018 | B/WAKAYAMA-C/50/2018 |
| B/FUKUI/23/2017 | B/OKAYAMA/20/2018 | B/OKINAWA/14/2018 |
| B/SAKAI/29/2017 | B/OKAYAMA/25/2018 | B/SHIZUOKA/28/2018 |
| B/NAGANO/2009/2018 | B/GIFU-C/2/2018 | B/YOKOHAMA/71/2018 |
| B/NAGANO/2778/2017 | B/YOKOHAMA/54/2017 | B/SAPPORO/25/2018 |
| B/AOMORI/8/2017 | B/YOKOHAMA/56/2017 | B/AICHI/129/2018 |
| B/KANAGAWA/ZC1722/2018 | B/YOKOHAMA/66/2017 | B/KANAGAWA/91/2018 |
| B/KANAGAWA/ZC1723/2018 | B/NIIGATA-C/23/2017 | B/AICHI/147/2018 |
| B/KANAGAWA/AC1722/2018 | B/TOKYO/17366/2017 | B/HYOGO/1245/2018 |
| B/KANAGAWA/IC1735/2018 | B/SAITAMA/194/2017 | B/AICHI/167/2018 |
| B/KANAGAWA/IC1736/2018 | B/MIE/19/2017 | B/MIYAGI/42/2018 |
| B/AICHI/113/2017 | B/MIE/21/2017 | B/SAGA/73/2018 |
| B/IWATE/36/2017 | B/YOKOHAMA/74/2017 | B/NAGANO/2199/2018 |
| B/KANAGAWA/AC1724/2018 | B/HIROSHIMA/8/2017 | B/YAMAGATA/171/2018 |
| B/KANAGAWA/AC1725/2018 | B/YOKOHAMA/80/2017 | B/FUKUI/52/2018 |
| B/KANAGAWA/IC1738/2018 | B/YAMANASHI/17240/2017 | B/KAWASAKI/127/2018 |
| B/KAGOSHIMA/74140/2017 | B/AICHI/68/2017 | B/AICHI/178/2018 |
| B/TOKYO/17539/2017 | B/SAITAMA/201/2017 | B/SAPPORO/36/2018 |
| B/SAITAMA/220/2017 | B/NAGANO/2732/2017 | B/YAMANASHI/18204/2018 |
| B/KANAGAWA/ZC1725/2018 | B/OSAKA-C/5/2017 | B/SENDAI/21/2018 |
| B/KANAGAWA/AC1728/2018 | B/YOKOHAMA/84/2017 | B/ISHIKAWA/63/2018 |
| B/KANAGAWA/IC1740/2018 | B/SAITAMA-C/22/2017 | B/TOCHIGI/18231/2018 |
| B/KANAGAWA/ZC1727/2018 | B/TOKYO/17436/2017 |  |

Table S4. Primers for the preparation of RNA controls

| Name | Sequence (5′ -3′) |
| --- | --- |
| **Primers for RT-PCR** | |
| PA-5ePF1 (for A/H1pdm) | AGCAAAAGCAGGTACTGAT |
| PA-3ePR1 (for A/H1pdm) | AGTAGAAACAAGGTACCTTTT |
| SZAPA-F (for A/H3) | CTCGAGCAAAAGCAGGTACTGAT |
| SZAPA-R2 (for A/H3) | AGTAGAAACAAGGTACYTTTTTGGAC |
| B-PA-F2 (for B) | AGCAGAAGCGRTRCGTTTGATTTGTC |
| B-PA-R4 (for B) | GAGTAGAAACACGTGCATTTTTGATTC |
| **Primers for Site-Directed Mutagenesis** | |
| H1pdm_PA/I38T(t113c)+ | CGAAACTAATAAGTTTGCTGCAACTTGCACACATTTGGAAGTTTG |
| H1pdm_PA/I38T(t113c)- | CAAACTTCCAAATGTGTGCAAGTTGCAGCAAACTTATTAGTTTCG |
| H3_PA/I38T(t113c)+ | ACCAACAAATTTGCAGCAACATGCACTCACTTGGAGG |
| H3_PA/I38T(t113c)- | CCTCCAAGTGAGTGCATGTTGCTGCAAATTTGTTGGT |
| B_PA/I38T(t113c)+ | CAGCAATGCTATTCAACACCTGTGTCCATCTAGAGGT |
| B_PA/I38T(t113c)- | ACCTCTAGATGGACACAGGTGTTGAATAGCATTGCTG |
| **Primers for PCR to prepare the template for *in vitro* transcription** | |
| H1pdm_PA-cds1F | ATGGAAGACTTTGTGCGAC |
| T7_H1pdm_PA-2151R | GATCACTAATACGACTCACTATAGGGCTACTTCAGTGCATGTGTGAG |
| H3_PA-cds1F | ATGGAAGATTTTGTGCGAC |
| T7_H3_PA-2151R | GATCACTAATACGACTCACTATAGGGCTATTTTAATGCATGTGTCAG |
| B_PA-CDS1F | ATGGATACTTTTATTACAAGAAAC |
| T7_B_PA-2181R | GATCACTAATACGACTCACTATAGGGTTATTCATCCATTATTTCATC |

Underlined sequence is T7 promoter.
